# Intestinal anti-inflammatory activity of *Ulva ohnoi* oil in DSS-induced experimental mouse model

**DOI:** 10.1038/s41598-021-94475-z

**Published:** 2021-07-23

**Authors:** JeongEun Kang, JiSun Park, Jung-Kil Seo, WonHyung Choi, SooCheol Choi, Ju-Hyoung Kim, In-Ah Lee

**Affiliations:** 1 Department of Chemistry, Kunsan National University, Gunsan, 54150 Korea; 2 Department of Food Science and Bitoechnology, Kunsan National University, Gunsan, 54150 Korea; 3 Department of Infection Biology, School of Medicine, Wonkwang University, Iksan, 54538 Korea; 4 Department of Aquaculture and Aquatic Science, Kunsan National University, Gunsan, 54150 Korea

**Keywords:** Biochemistry, Chemical biology

## Abstract

This study was conducted to examine the physiological activity of *Ulva ohnoi*, some of which may be used for food or natural products but could disturbing coastal ecosystems due to large scale green-tide, to check values of *U. ohnoi* oil through experimental results. *U. ohnoi* oil was extracted from bulk of *Ulva* biomass to confirm its antioxidant and antibacterial activity, and the efficacy of *U. ohnoi* oil in the state of inflammation was confirmed through animal experiments. To confirm the anti-inflammatory effect, a mouse model induced with DSS was used. As a result of measuring NO using plasma after induction of inflammation, the amount of NO produced in the *U. ohnoi* oil group was decreased compared to the control group. Expression of inflammatory cytokines TNF-α, IL-6, and IL-1β was decreased compared to the control group. As a result of observing H&E staining, lower crypt loss and inflammatory cell infiltration were found in the *U. ohnoi* oil group compared to the control group. Consequently, *U. ohnoi* oil appears to have great anti-inflammatory properties.

## Introduction

All living organisms produce reactive oxygen species (ROS), such as superoxide radical anion (O_2_^−^), hydrogen peroxide (H_2_O_2_), and hydroxyl radical (OH·), in the process of generating energy using oxygen^1^. ROS is also produced by external stimulation such as ozone and air pollution^2^. Oxidative stress caused by free radicals causes non-selective and irreversible modifications to the DNA, proteins, sugars, and lipids in living bodies, leading to aging and disease such as cancer, brain disease, heart disease, autoimmune disease, and arteriosclerosis^3,4^. Antioxidants are needed for protection against side effects because of the relative instability of free radicals and the possibility of damage to cells and tissues^5^. Therefore, many natural antioxidants, such as tocopherol and vitamin C, and synthetic antioxidants, such as dibutyl-1-hydroxytoluene (BHT) and butylhydroxyanisole (BHA), are used in response to such problems^6,7^. Synthetic antioxidants are effective and economical, but high doses are known to reduce phosphatide, blood enzymes, catalase, and peroxide^8–10^.

Tumor necrosis factor-α (TNF-α) is an inflammatory cytokine that was first discovered as an endotoxin-induced serum factor that causes necrosis of tumors in 1975 and is known as a derivative of inflammatory responses to various infections or tumors^11^. Interleukin-6 (IL-6) is a multifunctional cytokine produced by B cells, T cells, and fibroblasts and is involved in immune and inflammatory responses^12^. Interleukin-1β (IL-1β) is a potent inflammatory cytokine that is crucial for host-defense responses to infection and injury and regulates various physiological responses such as cell recruitment, appetite, sleep, and body temperature^13^. In macrophages, various stimuli such as cytokines, TNF-α, and lipopolysaccharide (LPS) activate NF-κB, a transcription factor of inflammatory responses, resulting in the expression of inducible nitric oxide synthase (iNOS) and cyclooxygenase-2 (COX-2) to produce nitric oxide (NO) and cause inflammation^14,15^. NO is synthesized from L-arginine by the activity of iNOS and performs various biological functions^16^, acting as a physiological regulator and signaling molecule. However, excessive production may contribute to tissue damage from stroke and ulcerative colitis^17^.

Omega-3 fatty acids are polyunsaturated fatty acids, namely, eicosapentaenoic acid (EPA) and docosahexaenoic acid (DHA), and are known to be effective against cardiovascular disease, dementia prevention, insulin resistance, and inflammation. As omega-3 fatty acids cannot be produced from the body and must be obtained from dietary sources^18^, extracting EPA and DHA from fish involves problems related to marine pollution^19^.

The Antarctic krill (*Euphausia superba*) is a zooplankton found in the Antarctic water that feeds directly on minute phytoplankton. This species is receiving the spotlight as a food resource in the future due to its excellent nutritional value^20^, as it contains large amounts of unsaturated fatty acids and antioxidants such as EPA and DHA^21^. However, krill contains a large amount of fluoride, which is harmful to the human body^22^. There are also reports that global warming is accelerating due to the decline in krill populations. Therefore, it is urgent to find a replacement because overfishing of krill threatens the survival of other marine life such as whales, penguins and seals^23^.

Seaweeds are known to accumulate many elements on from the surrounded environments. In particular, seaweeds absorb these elements over their entire surface area and are a treasure trove of minerals up to tens of thousands of times compared to pelagic organisms^24^. Although seaweeds have low nutritional value due to their low protein and fat content, they have antioxidant effects such as scavenging free radicals within the tissue^25,26^ due to their high content of various inorganic salts, such as potassium, iodine, and calcium, and vitamins A and C^27^. Recently, as the interest in finding substances with pharmacological action from natural plants has increased, efforts to extract effective active ingredients and use them as food additives or pharmaceuticals are growing alongside research on the physiological activity of sea weeds^28,29^.

*Ulva ohnoi* is a green alga in the family Ulvaceae (Chlorphyta) widely distributed around the North America, South Korea, Japan, and Mediterranean sea, usually attached to hard bottom on the rocky shore. The rise of sea surface temperature and nutritive salts due to global warming causes mass reproduction of *U. ohnoi*^30^, causing large scare green-tide in some contaminated costal ecosystem, such as Japan^31^, and South Korea^30^. *U. ohnoi* has a fast growth rate, so no problem of material depletion is raised, and it is thought that it is valuable as a new marine natural product while solving environmental problems. About 700 tons of *U. ohnoi* were collected in blooming area in Japan, and some algal biomass were used as food ingredients^31^. In addition, morphologically similar green alga, *U. lactuca* has been used for food in Europe for a long time, and multiple studies have been performed on its various food and physiological activities. It contains a large amount of polysaccharides containing sulfuric acid, and this acid polysaccharide has been reported to have anti-viral, immunity-boosting, and anti-cancer effects^32^. Some studies in South Korea reported anti-cancer effects and immune activities by extracting glycoproteins from single-cell layered green algal, *Monostroma nitidum*^32^.

The purpose of this study is to extract oil from *U. ohnoi* using an organic solvent extraction method to find out the utility of *U. ohnoi* oil for its physiological activity. There have been various efficacy studies using *U. ohnoi*, but studies using *U. ohnoi* oil are insufficient. Conventionally, oily ingredients such as krill oil and omega-3 are exerting the function of maintaining human health. We would like to consider the aspect of recycling discarded *U. ohnoi* through the use as a new material by proving the functionality of *U. ohnoi,* the subject of our research.

## Materials and methods

### Instruments and reagents

In this experiment, DPPH (1,1-diphenyl-2-picrylhydrazyl) (Sigma-Aldrich, USA), folin&ciocalteu’s reagent (sigma-Aldrich, USA), bacto agar (BD DIFCO, USA), bacto tryptic soy broth (BD DIFCO, USA), NO (nitric oxide) kit (Cell Biolabs. INC., USA), ELISA (enzyme-linked immunosorbent assay) kit (Abfrontier, KOR), TRIzol reagent (Sigma-Aldrich, USA), chloroform (Daejung, KOR), isopropyl alcohol (Sigma-Aldrich, USA), ethanol (Daejung, KOR), DEPC (diethyl pyrocarbonate) water (Thermo Scientific, USA), agarose (Bioneer, KOR), RT-PCR premix kit (Bioneer, KOR), Hematoxylin (Sigma-Aldrich, USA), Eosin Y (Sigma-Aldrich, USA ), centrifuge (Labogene, KOR), enzyme-linked immunosorbent assay (ELISA) reader (Thermo Scientific, USA), fluoro box (Neo Science, KOR), and Permount (Fisher, USA) were used.

### Experimental material

The *U. ohnoi* used in this study was collected from the north-east part of Jeju Island (A : Gujwa-eup, 33°33′33.0"N, 126°45′33.7", B : Jochon-eup, 33°32′57.9"N, 126°38′58.9"E, and C : Sinyang sand beach, 33°26′06.0"N, 126°55′23.4"E) in 2019 (voucher specimen number ELMV 03; deposited in the herbarium of ELMV laboratory in Kunsan National University; collector: JuHyoung Kim). We have transported small amount of Ulva biomass collected from the blooming area to the laboratory, thus sample collection was not threatened with endangered species and have not destroyed natural seaweed habitats. In addition, sampling was carried out after permission of National Research Foundation (NRF) of South Korea, thus government is permitting the collection of Ulva because it is working on a research project to manage Ulva biomass. Sample were washed with tap water and distilled water several times, and dried in an oven at 60℃. Organic solvent extraction was performed to extract *U. ohnoi* oil. The weight of the dried sample in the beaker was 29.3 g, 33.5 g, and 30.7 g of A, B, and C, respectively, and 200 ml of hexane was added per 10 g. Thereafter, extraction was performed at room temperature for 48 h and the organic solvent was removed using speed vacuum. The yields are 0.19%, 0.23%, and 0.21% for A, B, and C, respectively. All the experiments involving plants adhered to relevant ethical guidelines on plant usage. Krill oil is manufactured by Neptune (Neptune Technologies & Bioressources INC., Canada), and omega-3 is manufactured by Now Foods (Now Foods, USA). The composition of krill oil, omega-3, and *U. ohnoi* oil used in the experiment is shown in Table S1 of Supplementary Information.

### DPPH radical scavenging activity measurement

DPPH radical scavenging activity was used to eliminate absorption characteristics when the compound was stabilized by receiving electronic or hydrogen radicals and measured in accordance with using 1,1-diphenyl-2-picryhydrazyl (DPPH)^33^. DPPH was dissolved in ethanol at a final concentration of about 0.2 mM. *U. ohnoi* oil (100 µl) by concentration was added to 0.1 ml of ethanol solution of DPPH. After blocking the light and reacting for 20 min, the absorbance was measured at 517 nm. DPPH scavenging effect was calculated from [1—(*Abs*_*sample*_* – Abs*_*blank*_*)/Abs*_*control*_] × 100, where Abs_sample_ is the absorbance of the sample, and Abs_blank_ is the absorbance of the color control, and Abs_control_ is the absorbance of the negative control. As a control group, ethanol was added instead of the sample. L-ascorbic acid, which is known to have high antioxidant capacity, was used as a control to compare antioxidant activity.

### Total polyphenol content assay

The total polyphenol content assay (TPA) was determined using the Folin-Denis reagent^34^. 200 µl of the *U. ohnoi* oil was added to 1 ml of 0.2 N folin-ciocalteu reagent and 800 µl of 7.5% Na_2_CO_3_ was added. After blocking the light and reacting at room temperature for 2 h, the absorbance was measured at 620 nm using a spectrophotometer. Gallic acid was used as a standard reagent, and polyphenol content was calculated using a standard curve. The TPA was expressed as gallic acid equivalents (GAE) in mg/1 g material.

### URDA (ultrasensitive radical diffusion assay)

The antibacterial activity measurement using *U. ohnoi* oil was performed by URDA (ultrasensitive radical diffusion assay) method^35^. The sample was prepared at a concentration of 10 mg/ml using 0.01% acetic acid (HAc). The strains used were gram-positive bacteria *Bacillus subtilis* KCTC1021, gram-negative bacteria *Escherichia coli* D31, and fungus *Candidia albicans* KCTC76965. Each strain was inoculated with 1 platinum loop in tryptic soy broth (TSB) medium and pre-culture was performed at 37℃ for 24 h. The activated bacteria measured absorbance at 630 nm to make the O.D value of the bacteria 0.01 (≒ 0.5 McF). 0.03% TSB, sabouraud’s dextrose broth (SDB), 1% agarose, and 10 mM phosphate buffer (pH 6.5) were added to the underlay gel at 9.5 ml concentrations and then poured into the plate to solidify. The plate was pierced with a diameter of 2.5 mm and 5 µl of sample was injected into each well. All samples were tested with 0.01% acetic acid 5 µl, which was not affected by solvent. After the first culture at 37℃ for 3 h, 10 ml overlay gel containing 6% TSB, SDB, 1% agarose, and 20 mM phosphate buffer (pH 6.5) was poured into the cell, and then solidified at room temperature, and then the clear zone was confirmed after the second culture at 37℃ for 24 h.

### Characterization of antibacterial activators using enzymatic treatment

In order to confirm the properties of the antibacterial activity in the *U. ohnoi* oil, various enzymes were treated to determine whether there was any change in antibacterial activity. The enzymes used in the experiment are trypsin, chymotrypsin, lipase, α-amylase, pronase, and proteinase. 5 µl of *U. ohnoi* oil at a concentration 10 mg/ml was mixed with 1 µl of each enzyme and incubated for 3 h at 37 ℃, followed by secondary culture at 37 ℃ for 24 h. The cultured extracts were tested for URDA against *B. subtilis*, *E. coli*, and *C. albicans*.

### Experimental animals and inflammatory intestine disease induction

Male ICR mouse aged 4 weeks were obtained from Orient bio (Orient bio, Gwangju). They were housed in a controlled environment (25 ± 2 ℃, 50 ± 10% humidity) with a 12 h light/dark cycle. The animals allowed to free access to food and filtered water. They were quarantined and acclimatized for 1 week before use. The animals were kept in groups of 6 per cage. After 1 week habituation period, the experimental animals were classified into five groups as follows: one is normal, another is control and the others is DSS + krill oil (KO), DSS + omega-3 (ω-3), and DSS + *U. ohnoi* oil (UO). Krill oil, ω-3, and *U. ohnoi* oil are prepared using distilled water at concentrations of 100 mg/kg, 100 mg/kg, and 25 mg/kg and administered orally at 100 µl each for 10 days (Table S2). *U. ohnoi* oil used for mouse administration was prepared by mixing a certain amount of A, B, and C. To induce inflammatory bowel disease, the Con, KO, ω-3, and UO groups freely ingested 5% dextran sulfate sodium (DSS) dissolved in distilled water for 7 days after 4 days of sample administration. The body weight of the experimental animals was measured at the same time every day and is shown in Figure S1 of Supplementary Information. The animal studies were approved by Kunsan National University animal experiment ethics committee (Approval No: 2020-02). This study was carried out in compliance with the Animal Research: Reporting of In Vivo Experiments (ARRIVE) guidelines. All the experiments involving animals adhered to relevant ethical guidelines on animal usage.

### Measurement of NO production

The amount of NO production was measured using plasma of 6 mice per group, and Griess reagent was used^36^. Blood was collected to obtain plasma and centrifuged at 7000 rpm for 10 min at 4 ℃. NO concentration was then determined by the addition of 50 µl each Griess reagents 1 and 2. After incubation for 10 min at room temperature, the absorbance was read at 540 nm.

### Measurement of inflammatory cytokine in colon tissue

In this study, colon tissue from 6 mice per group was removed and used, 1 ml of 1 × phosphate-buffered saline (PBS) was added, crushed, and centrifuged at 7000 rpm for 10 min, and then the supernatant was taken. The amount of inflammatory cytokine TNF-α, IL-6, and IL-1β was measured using an enzyme-linked immunosorbent assay (ELISA) kit. After adding 100 µl of antigen to the antibody-coated plate, incubated at 37℃ for 2 h, and washed 3 times using a washing buffer. After the addition of 100 µl of the secondary antibody, it was incubated at 37℃ for 1 h and washed 3 times. After adding 100 µl of the TMB solution, incubate at 37℃ for 30 min, and reacted at room temperature for 10 min after adding 100 µl of stop solution, absorbance was measured at 450 nm.

### RT-PCR

RNA used in the experiment was extracted from the colon tissue of the mouse using TRIzol reagent, and it was dissolved in 50 µl diethyl pyrocarbonate (DEPC) water. Reverse transcription-polymerase chain reaction (RT-PCR) is performed using and RT-PCR premix kit, and 300 ng of total RNA, 20 pmol of each of forward and reverse primers are added to use a total of 20 µl of the reaction solution. The cDNA was synthesized by reacting at 50℃ for 30 min and 94℃ for 5 min, followed by 35 cycles of denaturation at 94℃ for 40 s, annealing at 58℃ for 40 s, and extension at 72℃ for 90 s. The PCR product was confirmed by electrophoresis on 1.2% agarose gel, and GAPDH was used as control. The primer sequence used in the experiment is as follows Table 1.

**Table 1:** Sequences of primers used for RT-PCR.

Gene	Forward/reverse	Sequences (5’ to 3’)
TNF-α	F	GGCAGGTCTATTTGGAGTCATTGC
	R	ACATTCGAGGTCCAGTGAATTCGG
IL-6	F	TGGAGTCACAGAAGGAGTGGCTAAG
	R	TCTGACCACAGTGAGGAATGTCCAC
IL-1β	F	GCCTTGGGCCTCAAAGGAAAGAATC
	R	GGAAGACACAGATTCCATGGTGAAG
COX-2	F	GCAAATCCTTGCTGTTCCAATC
	R	GGAGAAGGCTTCCCAGTTTTG
GAPDH	F	CATGGCCTTCGTGTTC
	R	CCTGGTCCTCAGTGTAGC

### H&E staining using colon tissue

Hematoxylin & eosin (H&E) staining is primarily used in histology to visualize the structure of tissues^37^. H&E stains are still essential for recognizing various tissue types and the morphologic changes that form the basis of contemporary cancer diagnosis^38^. Each specimen was sectioned into a thickness of 7 µm and histochemical staining was performed using hematoxylin and eosin. For deparaffinization, xylene was added and repeated three times for 5 min, and xylene was removed in the order of 100%, 95%, and 70% ethanol. For nuclear staining, it was stained with hematoxylin solution for 5 min, and then washed with flowing water. Hematoxylin was removed using 1% HCl solution and 1% ammonia solution, followed by immersion in eosin solution for 2 min to stain the cytoplasm. Then, it was dehydrated in the order of 70%, 95%, and 100% ethanol. To remove alcohol, it was put in xylene and repeated three times for 3 min, and then the cover glass was and sealed.

## Results and discussion

### Antioxidant activity evaluation

**Figure 1:**
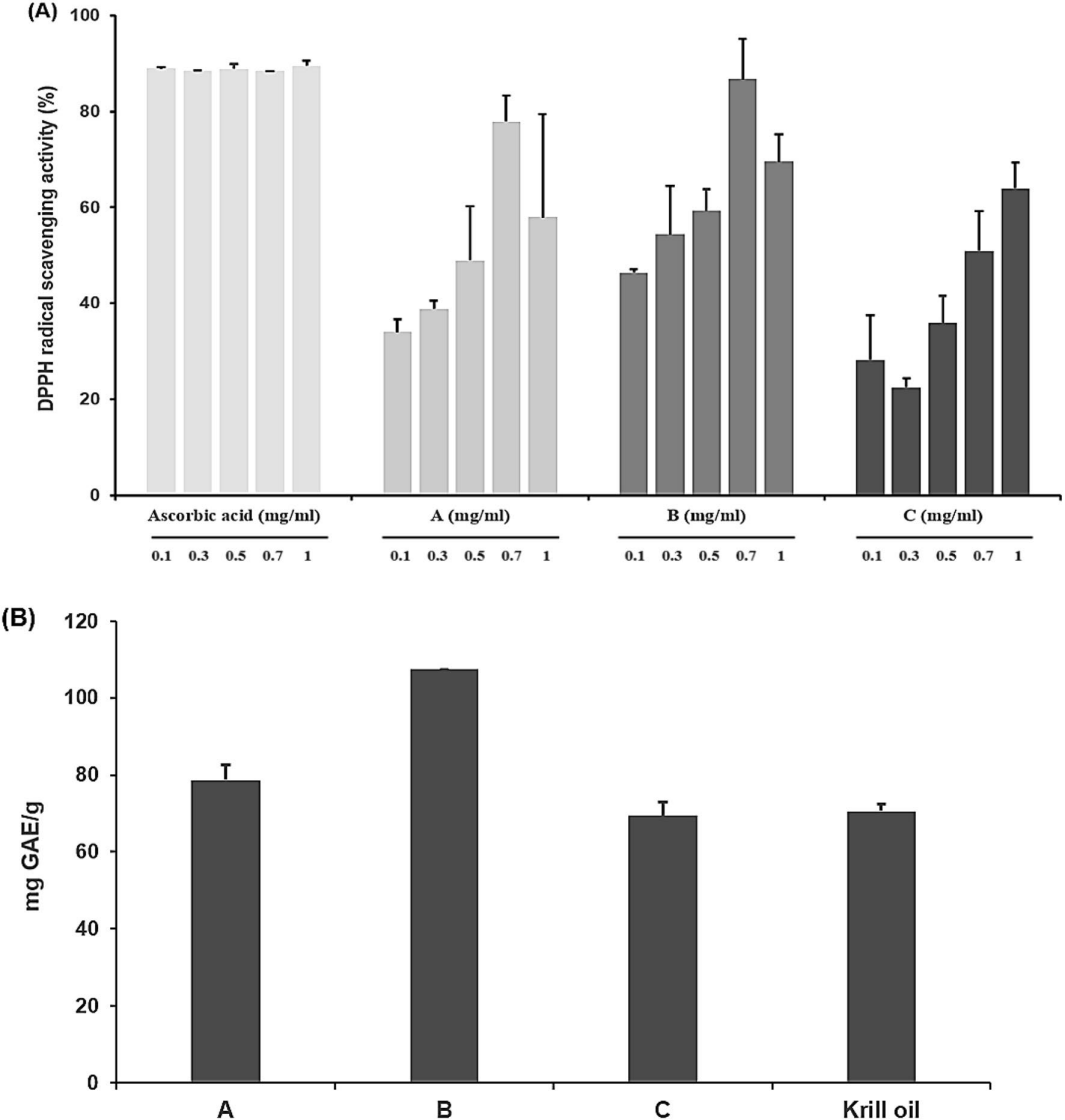
**(A)** DPPH radical scavenging activity of regional *U. ohnoi* oil at various concentrations. **(B)** Total polyphenol content of *U. ohnoi* oil by region.

The antioxidant activity of *U. ohnoi* oil was measured using DPPH and Folin-Denis reagent. The radical scavenging ability was measured by concentrations of 0.1, 0.3, 0.5, 0.7, and 1 mg/ml, and ascorbic acid was used as a positive control (Fig. 1A). It was confirmed that the DPPH radical scavenging activity of A and B *U. ohnoi* oil increased to 0.7 mg/ml, but decreased at the concentration of 1 mg/ml. At the concentration of 0.7 mg/ml, B *U. ohnoi* oil showed the most similar value to ascorbic acid at 86.71 ± 8.36%. Ascorbic acid showed 88% scavenging activity independent of concentration.

The total phenolic compound content of the sample was curve using gallic acid as the standard and converted to gallic acid equivalents (GAE) (Fig. 1B). The total polyphenol content was 107.45 ± 0 mg GAE/g in B and 69.44 ± 3.46 mg GAE/g in C, which was about 1.5 times higher than that of B *U. ohnoi* oil. Krill oil showed a lower polyphenol content than B *U. ohnoi* oil at 70.6 ± 1.99 mg GAE/g.

### Antibacterial activity of *U. ohnoi* oil by strain

The antibacterial activity of *U. ohnoi* oil was measured against *B. subtilis, E. coli*, and *C. albicans* strains using the URDA method. In general, *B. subtilis* and *E. coli* showed antibacterial activity, but not against the fungus *C. albicans*. The size of the clear zone was 10.36 mm in B and 7.35 mm in A in the *B. subtilis* strain, indicating that the clear zone size was 1.41 time higher than that of B *U. ohnoi* oil. As a result of measuring the activity against *E. coli* strains, all three types of U. ohnoi oil showed high antibacterial activity of 10 mm or more (Table 2A).

**Table 2:** (A) Size of clear zone by *U. ohnoi* oil. (B) Size of clear zone by *U. ohnoi* oil after with treatment enzyme (*NT* HAc 0.01% acetic acid, *T* trypsin, *C* chymotrypsin, *L* lipase, *α* α-amylase, *P* pronase, *K* proteinase K) (unit of measurements: mm).

(A)								
Bacterial strain	Region	Clear zone (mm)						
*B. subtilis*	A	7.35						
	B	10.36						
	C	9.73						
*E. coli*	A	12.38						
	B	12.25						
	C	10.36						
*C. albicans*	A	–						
	B	–						
	C	–						

(B)								
		NT	T	C	L	Α	P	K
*B. subtilis*	A	10.69	11.44	9.42	10.82	9.70	10.19	9.56
	B	10.57	9.06	8.73	8.83	8.33	8.77	8.64
	C	9.17	8.36	9.19	9.12	8.70	9.15	9.90
*E. coli*	A	14.67	14.33	14.00	13.83	13.38	13.57	14.21
	B	11.02	10.27	9.46	9.89	10.00	10.45	10.32
	C	12.22	10.98	11.61	9.41	8.98	9.63	9.98
*C. albicans*	A	–	–	–	–	–	–	–
	B	–	–	–	–	–	–	–
	C	–	–	–	–	–	–	–

### Changes in antibacterial activity by enzyme treatment

To confirm the characteristics of substances exhibiting antibacterial activity in *U. ohnoi* oil, six enzymes such as protease and lipolytic enzyme were treated, respectively, and then it was confirmed whether there was any change in antibacterial activity. As enzymes, trypsin, chymotrypsin, lipase, α-amylase, pronase, and proteinase K were used, and the size of the clear zone was measured to show antibacterial activity according to the enzyme treatment (Table 2B). *B. subtilis, E. coli*, and *C. albicans* strains were tested for activity changes, but the antibacterial activity of *U. ohnoi* oil was not significantly affected by the enzymes used. Therefore, it was confirmed that the substance exhibiting antibacterial activity in *U. ohnoi* oil is considered to an organic compound rather than a fat or proteinaceous substance that is cleaved by enzyme treatment and has stability in enzyme reaction.

### NO production measurement

**Figure 2:**
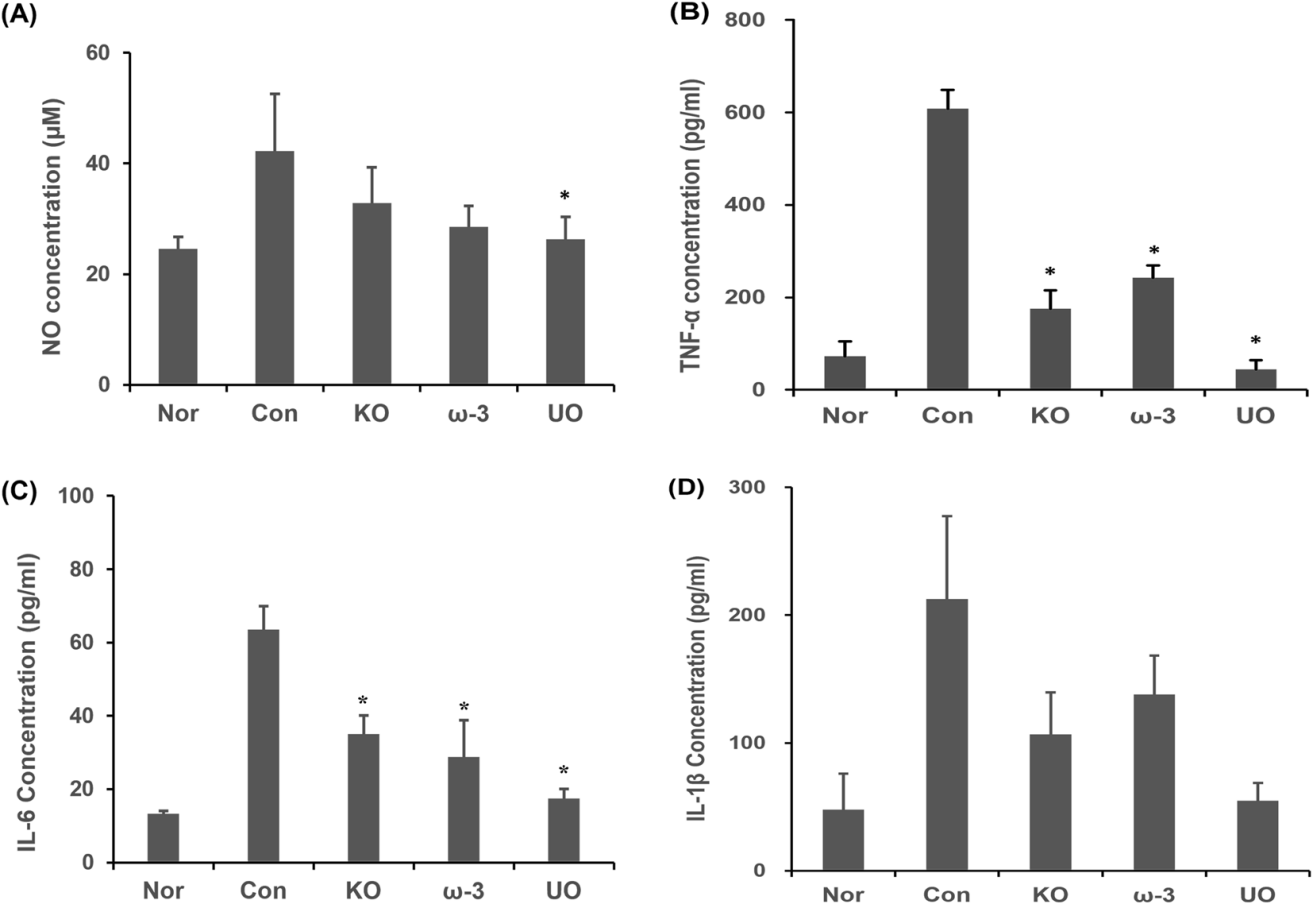
**(A)** Effect of *U. ohnoi* oil on nitric oxide production in mouse plasma. Data obtained for group were analyzed ANOVA compared control group. Significantly different from control group (*p < 0.05). Effect of *U. ohnoi* oil according to the **(B)** TNF-α, **(C)** IL-6, and **(D)** IL-1β production in the colon tissue of mice induced by DSS. Data obtained for group were analyzed ANOVA compared control group. Significantly different from control group *p < 0.05, n = 6.

NO (nitric oxide) is known as a mediator of the inflammatory response secreted when cells stimulated by inflammatory substances such as LPS or TNF-α induce inflammation^39^. In this study, the amount of NO production was measured using plasma of mice (Fig. 2A). The NO production of the control group was 31.26 ± 5.04 µM, which was about 1.3 times higher than that of the normal group, and it was found that there is an effect of inducing inflammation by DSS. The production amount of the UO group was 26.26 ± 4.08 µM, which was about 84.0% lower than that of the control group, and it was confirmed that the production amount of NO was like that of the normal group.

### Inflammatory cytokine production

The amount of TNF-α, IL-6, and IL-1β production were measured by ELISA method. All three cytokines were increased in the control group. In the case of TNF-α and IL-6, the amount of production of 43.9 ± 20.2 pg/ml and 17.41 ± 2.68 pg/ml, respectively, in the UO group and significantly decreased compared to the control group (Fig. 2B,C). The amount of IL-1β produced in the UO group was 54.68 ± 14.14 pg/ml, which was like that of the normal group, but there was no statistical significance (Fig. 2D).

### Confirmation of inflammatory cytokine mRNA expression

**Figure 3:**
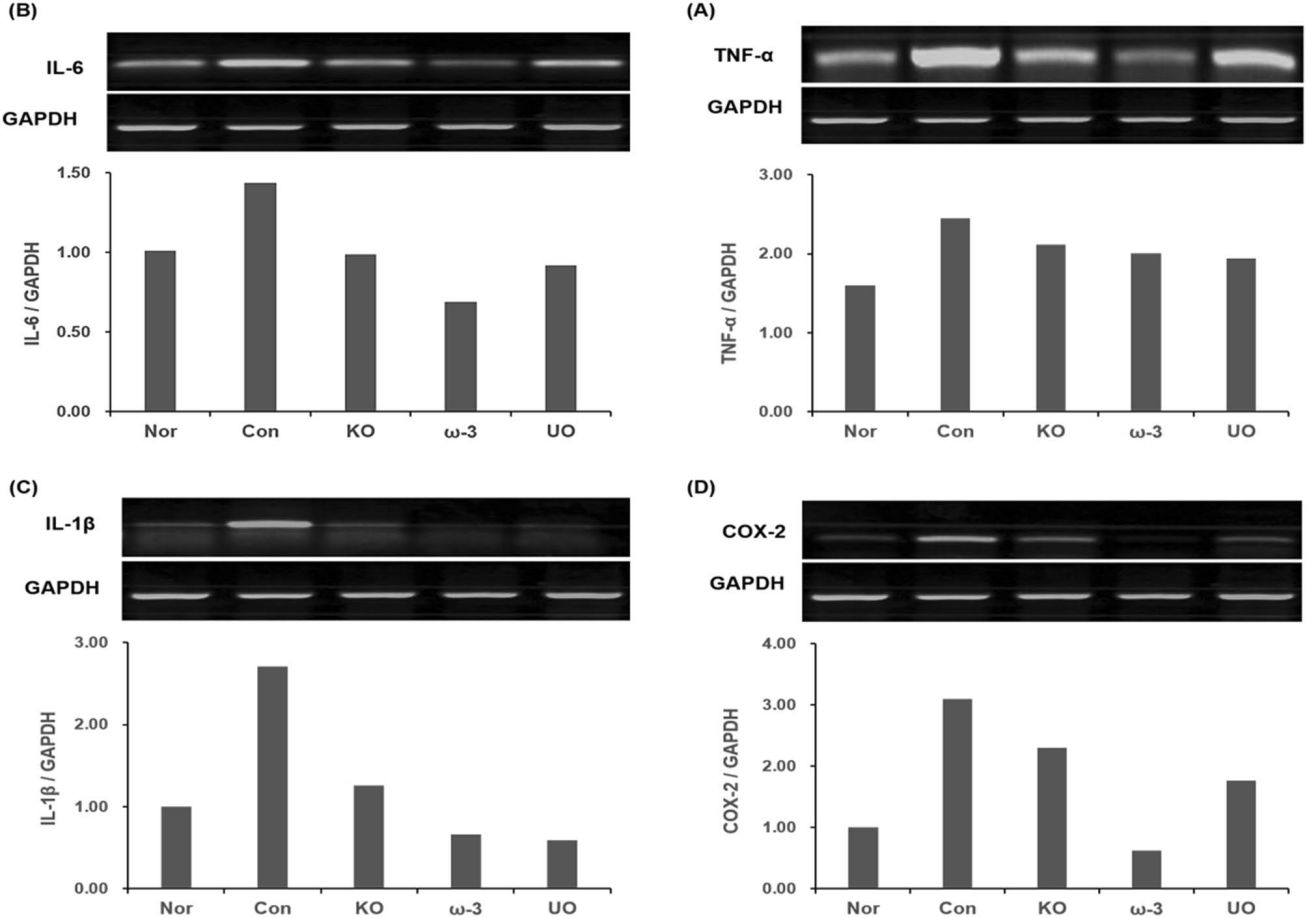
Inhibitory effects of *U. ohnoi* oil extracts on the mRNA expression of inflammatory cytokine. GAPDH was used as an internal control. **(A)** TNF-α/GAPDH, **(B)** IL-6/GAPDH, **(C)** IL-1β/GAPDH, and **(D)** COX-2/GAPDH were quantified by a numerical graph. n = 6.

RT-PCR was performed using the colon tissue of experimental animals induced with 5% DSS to determine whether inflammatory cytokine affects mRNA expression. The results are as follows (Fig. 3). When compared with the normal group, it was confirmed that the amount of inflammatory factor expression in the control group was increased. It was confirmed that the expression amount of the *U. ohnoi* oil administration group decreased compared to the control group, but there was no significant difference in the case of TNF-α.

### Histochemical observation

**Figure 4:**
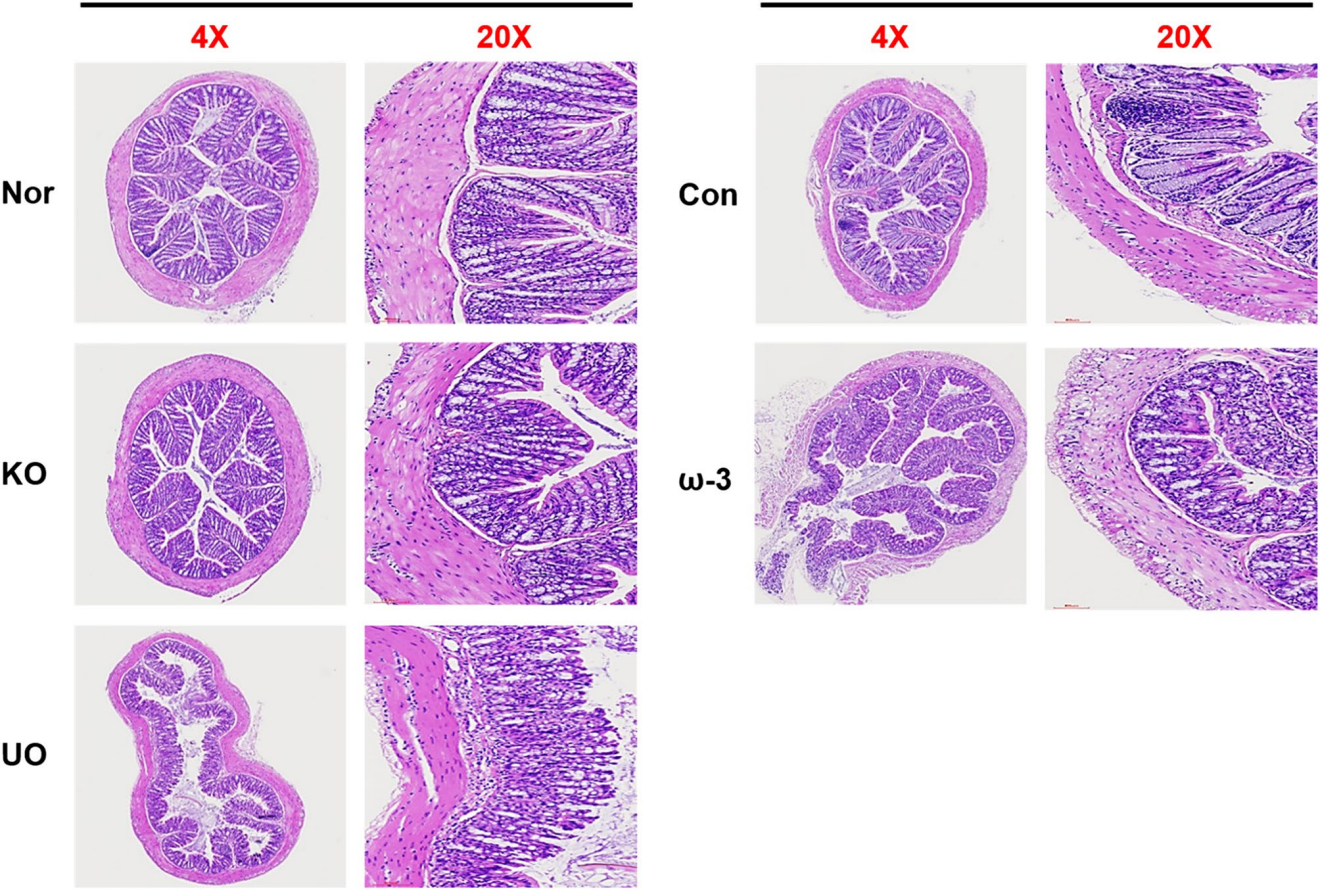
The colon tissue was section to a thickness of 7 µm and stained with H&E. Histologic observation of the intestine induced by DSS. Normal: without of DSS, Control: DSS-induced colitis, KO: DSS and krill oil 100 mg/kg administration, ω-3: DSS and ω-3 100 mg/kg administration, and UO: DSS and *U. ohnoi* oil 25 mg/kg administration. n = 6.

The histological examination of the intestine was evaluated for morphological changes through hematoxylin and eosin (H&E) staining, and observed using an optical microscope. The normal group represents typical colon tissue, but the control group lost crypt and destroyed the nucleus (Fig. 4). On the other hand, in the *U. ohnoi* oil-administered group, submucosal infiltration was observed compared to the normal group, but the degree of crypt and tissue damage was lower than that of the control group.

## Discussion

This study recognized the superiority of *U. ohnoi*, a species of green algae known for its immune activity, based on the results of prior research using *U. ohnoi* extracts to investigate its effects on physiological activity. This study conducted experiments to evaluate the antioxidant, antibacterial activities of the *U. ohnoi* oil, and measured its anti-inflammatory activity by using a DSS-induced inflammatory bowel disease mouse model. As a result of measuring DPPH radical scavenging activity to evaluate the antioxidant activity of *U. ohnoi* oil, the radical scavenging activity of A and B *U. ohnoi* oil was 77.85 ± 5.39% and 86.71 ± 8.36%, respectively, at 0.7 mg/ml, showing the highest activity. In particular, B *U. ohnoi* oil showed similar activity to the control substance, ascorbic acid. When the total polyphenol was measured, it was confirmed that among the three types of *U. ohnoi* oil, *U. ohnoi* oil of B contained the highest polyphenol as 107.45 ± 0 mg GAE/g. This confirms that *U. ohnoi* oil exhibits a function that can replace krill oil, an animal oil. In addition, the efficacy of antioxidants was repeatedly confirmed through additional experiments. Since the existing oils with excellent antioxidant efficacy are being used in abundance in the food and cosmetics industry, it is possible to industrially use *U. ohnoi* oil, which has antioxidant properties.

Meanwhile, as a result of evaluating the antibacterial activity of *U. ohnoi* oil using URDA method, *B. subtilis* and *E. coli* strains showed strong antibacterial activity, but no antibacterial activity was observed for *C. albicans*. In the *B. subtilis* strain, the *U. ohnoi* oil from B showed higher activity than the *U. ohnoi* oils from other regions, and there was no significant difference in the *C. albicans* strain. The change in antibacterial activity was measured by treating six enzymes to investigate the properties of the antibacterial active ingredients of *U. ohnoi* oil, but there was no significant change compared with before treating enzymes. Ulva ohnoi oil showed some antibacterial activity, and this is a result that can prove the wide utility of U. ohnoi oil. In addition, it can be suggested as one of the many causes of reduced inflammation in the research results related to colitis.

After inducing inflammatory bowel disease with DSS, this study examined the anti-inflammatory effect by administering krill oil, ω-3, and *U. ohnoi* oil. Compared with the normal group, weight loss and diarrhea were observed in all groups administered with DSS. In terms of proof, the *U. ohnoi* oil administered group and the normal group produced a similar amount of NO (an inflammation mediator) compared to the control group, indicating the effect of *U. ohnoi* oil to inhibit NO production. The inhibition of production of TNF-α IL-6, and IL-1β of *U. ohnoi* oil was measured through ELISA. The production of cytokines increased significantly the control group compared to the normal group, and the production was inhibited in the *U. ohnoi* group. The exact mechanism for the effectiveness of Ulva ohnoi oil to inhibit inflammatory cytokines will need to be investigated through future studies.

In case of TNF-α and IL-6, the *U. ohnoi* oil group showed a significant decrease compared to the control group. In case of the mRNA expression of inflammatory factors (TNF-α, IL-6, IL-1β, and COX-2) in the colon tissue, the *U. ohnoi* oil group showed similar expression levels to the krill oil administered group. These results support the results of measuring inflammatory cytokines in colon tissue.

As a result of analyzing the inflammatory response of the colon tissue using H&E staining, the group that induced bowel disease with DSS showed crypt loss and inflammatory cell infiltration, but this was mitigated in the mice treated with DSS *U. ohnoi.* These results show that *U. ohnoi* oil has a higher anti-inflammatory effect than krill oil in mice with DSS-induced inflammatory bowel disease, and that it was effective in inhibiting inflammatory expression.

Krill oil, which has previously been helping health, needs an alternative material due to the decrease in krill shrimp. It is similar to the efficacy of krill oil, which is the *U. ohnoi* oil used in this study, and the one-minute efficacy is more beneficial. The *U. ohnoi* is being multiplied due to the warming of the climate, and the part that can be used in the industry is extremely weak. As the result of this study, it is expected that if the usefulness of *U. ohnoi* oil is utilized in industry, there will be a synergistic effect of reducing environmental pollution and utilizing biomass.

### Statistical analysis

Data were analyzed using the predictive analytics software (PASW 18 for Windows) and presented as mean ± S.D. A one-way analysis of variance (ANOVA) test was performed to assess the NO production in mouse plasma and the generation of inflammatory cytokines in the colon. The significance level was set at ^*^p < 0.05.

## Supplementary Information

Supplementary Information.

## Data Availability

The datasets used and/or analyzed during the current study are available from the corresponding author upon reasonable request.
