# Supplementary material for: Intestinal anti-inflammatory activity of Ulva ohnoi oil in DSS-induced experimental mouse model
Source: Sci Rep. 2021 Jul 23;11:15087. doi: 10.1038/s41598-021-94475-z (PMC8302574; doi:10.1038/s41598-021-94475-z)
Supplement: Supplementary file 1 — Supplementary Information. [file 41598_2021_94475_MOESM1_ESM.docx]

**Supplementary Information**

(A) krill oil

|  | Per 1 g of krill oil |
| --- | --- |
| Vitamin A | 100 IU |
| Vitamin E | 0.5 IU |
| Choline | 73 mg |
| Omega-3 | 300 mg |
| Omega-6 | 20 mg |
| Astaxanthin | 1.5 mg |

(B) omega-3

|  | Per 2 g of Fish oil concentrate |
| --- | --- |
| Eicosapentaenoic Acid (EPA) | 360 mg |
| Docosahexaenoic Acid (DHA) | 240 mg |

(C) *U. ohnoi* oil

| No | RT | Compounds |
| --- | --- | --- |
| 1 | 7.275 | 2,4-Dimethyl-heptane |
| 2 | 13.072 | 3-Hexanol |
| 3 | 17.712 | Tetradecane |
| 4 | 27.677 | 2,6,11-Trimethyl-dodecane |
| 5 | 33.096 | Heptacosane |
| 6 | 39.230 | Heneicosane |
| 7 | 45.743 | 2,6,11,15-Tetramethyl-hexadecane |
| 8 | 53.140 | Tricosane |
| 9 | 55.424 | Tetracosane |
| 10 | 58.254 | Pentacosane |

**Table S1.** (A) Composition of krill oil used in the experiment. (B) Composition of omega-3 used in the experiment. (C) B *U. ohnoi* oil was analyzed through GC-MS (Gas Chromatography-Mass Spectrometry). The retention time and compounds of the main peak are shown.

| (n=6) | 5% DSS | Extract |
| --- | --- | --- |
| Nor | - | - |
| Con | + | - |
| KO | + | Krill oil 100 mg/kg |
| ω-3 | + | ω-3 100 mg/kg |
| UO | + | *U. ohnoi* oil 25 mg/kg |

**Table S2.** The scheme of *in vivo* study.


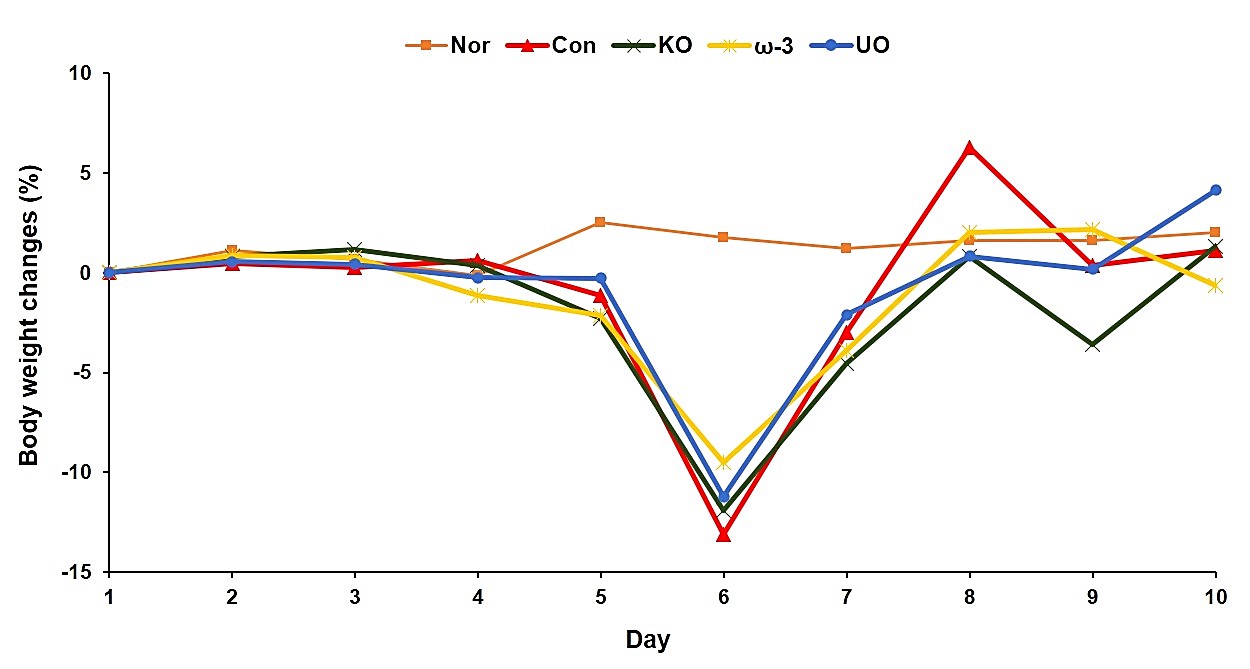


**Fig S1.** Percent change in body weight over time. Experimental colitis in mice (n=6 / group) was induced by a 5% DSS dissolved in the drinking water for 7 days. KO (100 mg/kg), ω-3 (100 mg/kg), and UO (25 mg/kg) was oral administered once a day for 10 days. In order to induce inflammatory bowel disease, all groups except the normal group were allowed to freely drink 5% DSS (dextran sulfate sodium) dissolved in distilled water for 7 days from the 4th day of sample administration, and the normal group consumed water.


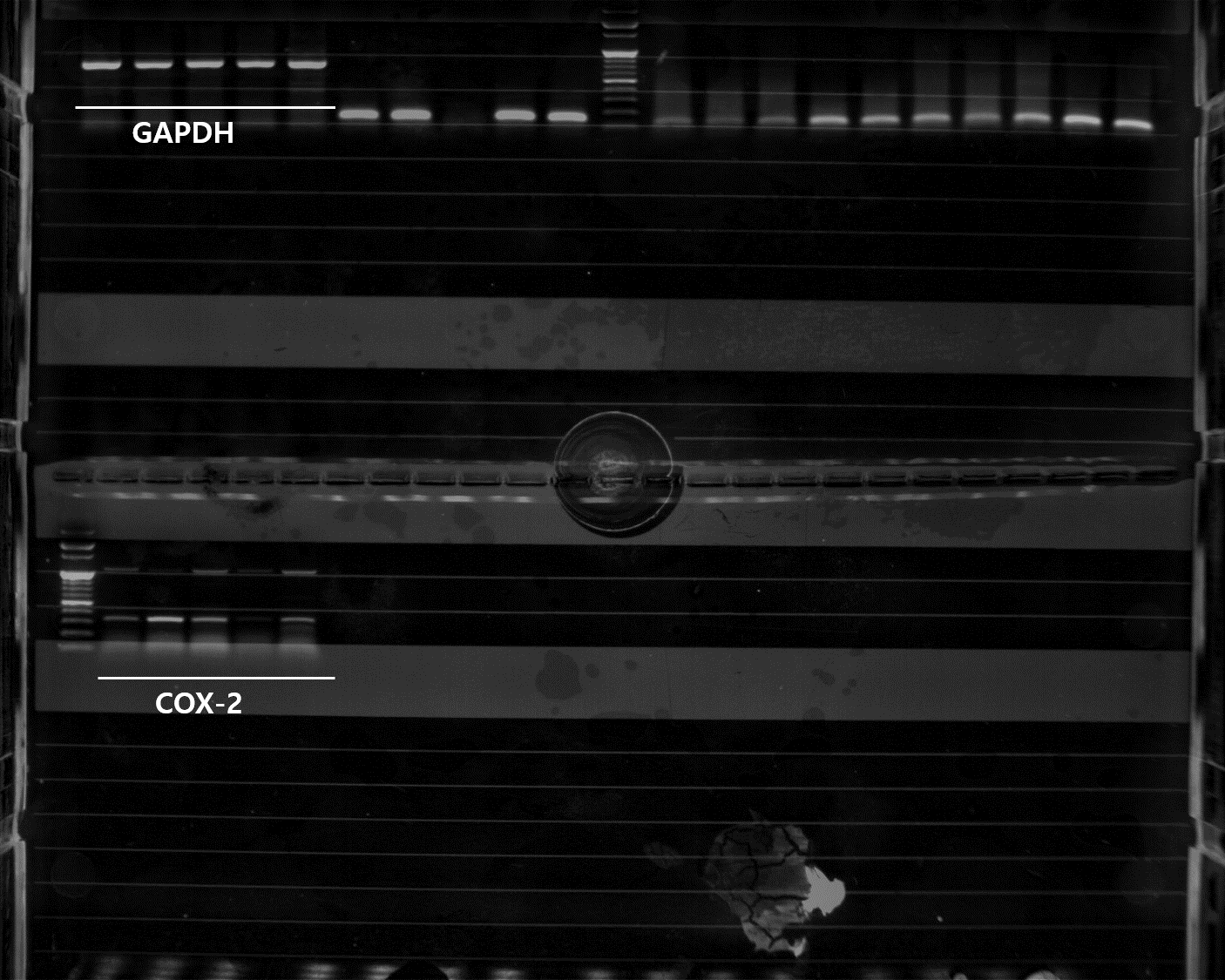


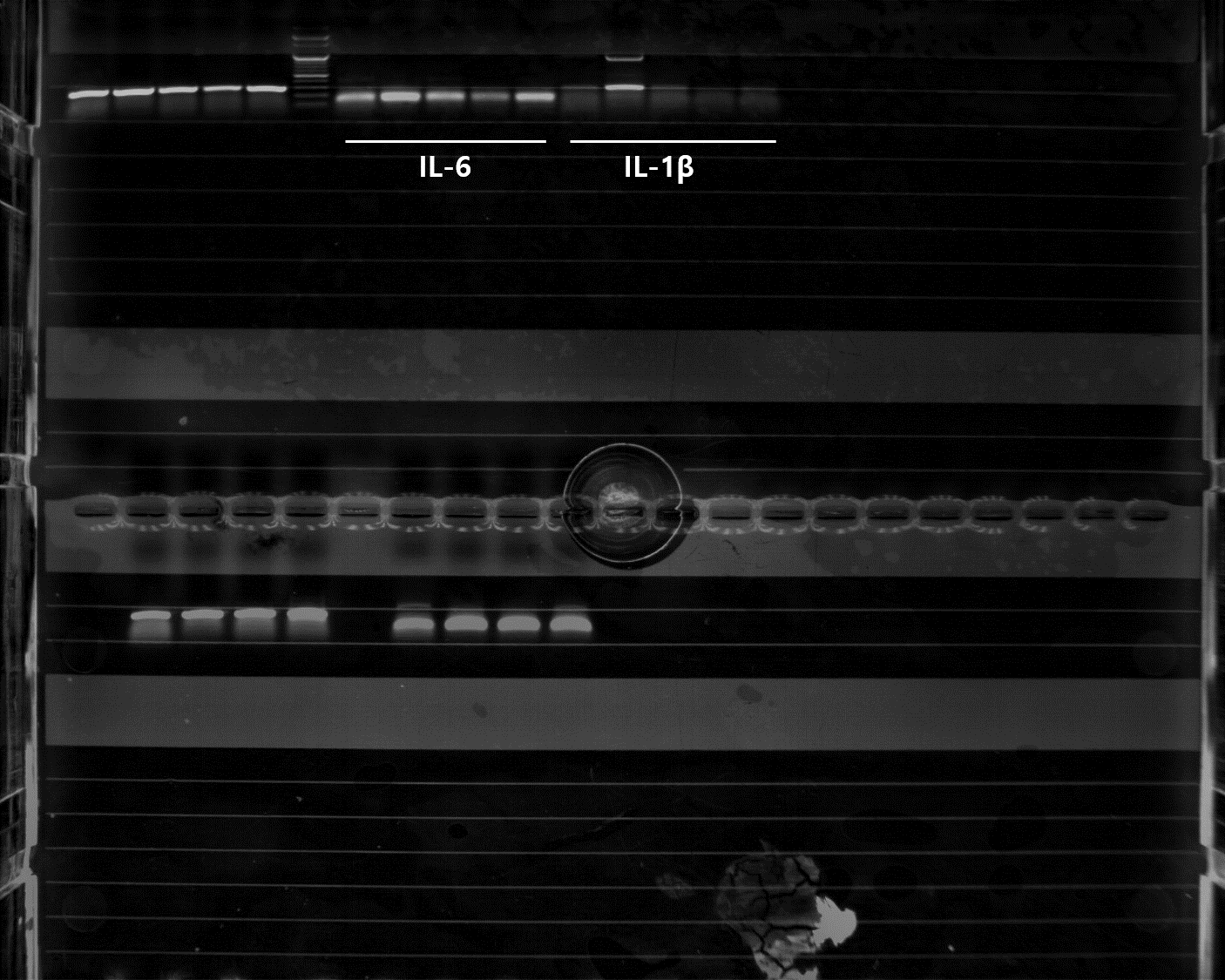

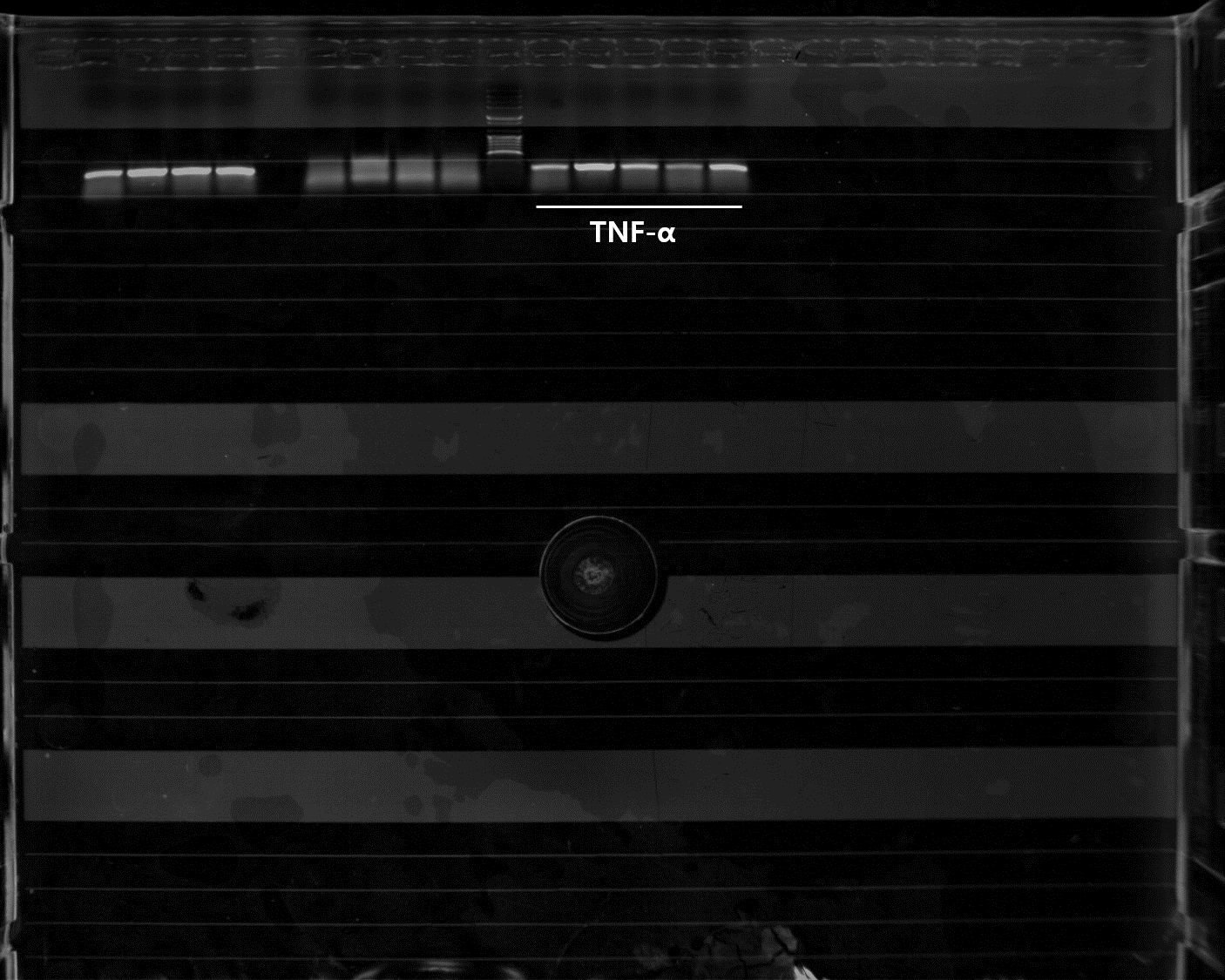


**Fig S2.** Original file for Manuscript Fig. 3

**Method**

***LPS-induced RAW 264.7 cell inflammation model***

LPS is known to be a representative pathogenic substance that induces a macrophage-mediated inflammatory response. In this study, RAW 264.7 cells were aliquoted into a 24-well plate in 2 × 10^5^ cells and cultured for 24 hours. 1 μg/ml of LPS and U. ohnoi oil were treated with 0.1, 0.5, and 1 mg/ml, incubated for 24 hours in 37℃, 5% CO_2_ incubator, and then the supernatant was removed and washed twice with 1x PBS. Thereafter, RNA was extracted from the cells using TRIzol reagent was dissolved in DEPC-water and used. Reverse transcription-polymerase chain reaction (RT-PCR) is performed using and RT-PCR premix kit, and 300 ng of total RNA, 20 pmol of each of forward and reverse primers are added to use a total of 20 ㎕ of the reaction solution. The cDNA was synthesized by reacting at 50℃ for 30 minutes and 94℃ for 5 minutes, followed by 35 cycles of denaturation at 94℃ for 40 seconds, annealing at 58℃ for 40 seconds, and extension at 72℃ for 90 seconds. The PCR product was confirmed by electrophoresis on 1.2% agarose gel, and β-actin was used as control. The primer sequence used in the experiment is as follows Table 1.

| Gene | Forward /  Reverse | Sequences (5’ to 3’) |
| --- | --- | --- |
| iNOS | F | CACCTTGGAGTTCACCCCAGT |
|  | R | ACCACTCGTACTTGGGATGC |
| Catalase | F | GTCGTCTCCTTGCTTTTTGC |
|  | R | TCTGCTCGAAGTGAATGACG |
| β-actin | F | GTGGGCCGCTCTAGGCACA |
|  | R | CGGTTGGCCTTAGGGTTCAGG |

**Table S3.** Sequences of primers used for RT-PCR.

***Myeloperoxidase (MPO) activity***

The colon tissue was subjected to ultrasonic grinding by adding 1 ml of 50 mM sodium phosphate buffer (pH 6.0) containing 0.5% hexadecyl trimethyl ammonium bromide, followed by centrifugation at 12000 rpm and 4℃ for 10 minutes, and the supernatant was used as an enzyme solution. After adding 30 ㎕ of O-dianisidine (20 mg/ml) to 100 ㎕ of the enzyme solution, H_2_O_2_ was added to a final concentration of 0.0005%, followed by reaction at 20℃ for 10 minutes. After stopping the reaction by adding 30 ㎕ of 2% sodium azide, the enzyme activity was measured with a Multiskan GO Microplate Reader at 25℃ and 460 nm. MPO activity 1 unit represents the activity of an enzyme that degrades H_2_O_2_ 1 μmole/min at 25℃, and is expressed as MPO unit per gram of tissue.

**Results**

***Confirmation of mRNA expression through LPS induction***

After LPS treatment in RAW 264.7 cells, the expression levels of the inflammatory cytokine iNOS and the antioxidant enzyme catalase were measured (Fig.S3). In the case of iNOS, it was confirmed that the expression was suppressed in a concentration-dependent manner when *U. ohnoi* oil was treated. As a result of measuring the expression level of catalase, which is an antioxidant-related factor, it was found that the LPS decreased compared to the normal. In addition, in the group treated with 1 mg/ml of U. ohnoi oil, an activity similar to that the normal group was confirmed.


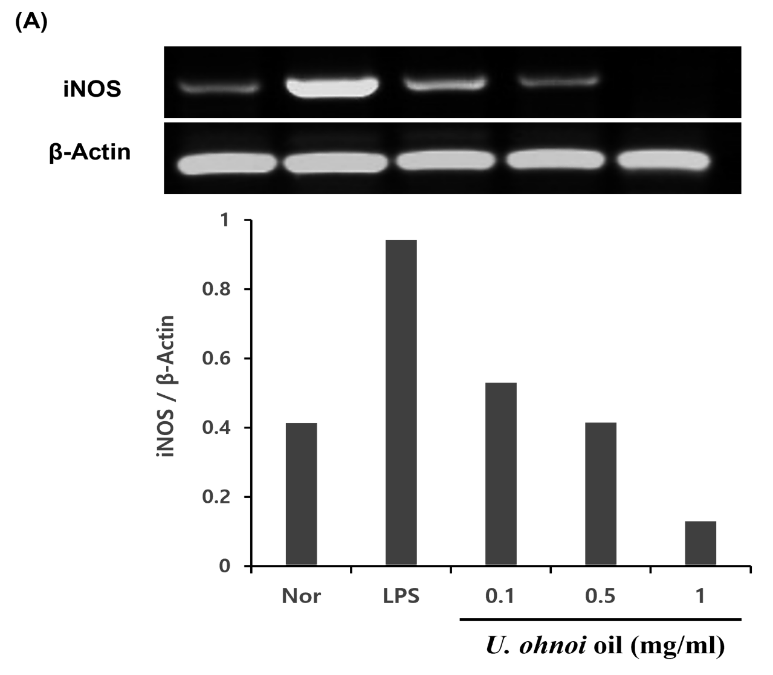


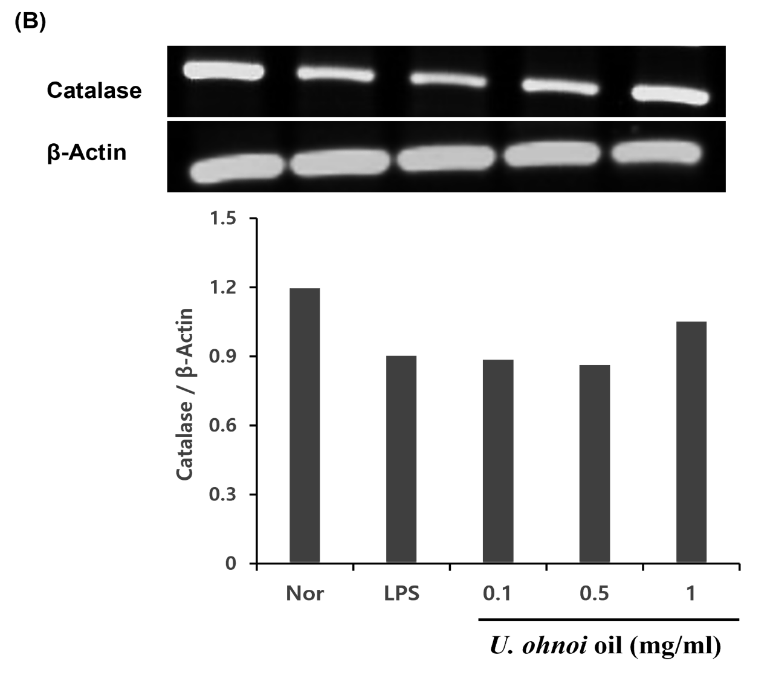


**Fig S3.** Inhibitory effect of U. ohnoi oil on iNOS and Catalase on mRNA expression. β-actin was used as an internal control. (A) iNOS/β-actin, (B) Catalase/β-actin were quantified by a numerical graph. n=6.

***Evaluation of MPO inhibition of U. ohnoi oil using colon tissue***

One of the inflammatory reactions of the tissue is the penetration of neutrophils into the tissues. To measure the degree of neutrophil penetration, the activity of MPO, an enzyme that is highly expressed in neutrophils in the colon tissue, was measured (Fig. S4). MPO activity significantly increased in the control group compared to the normal group, and the activities in the KO, ω-3, and UO groups were decreased compared to the control group.


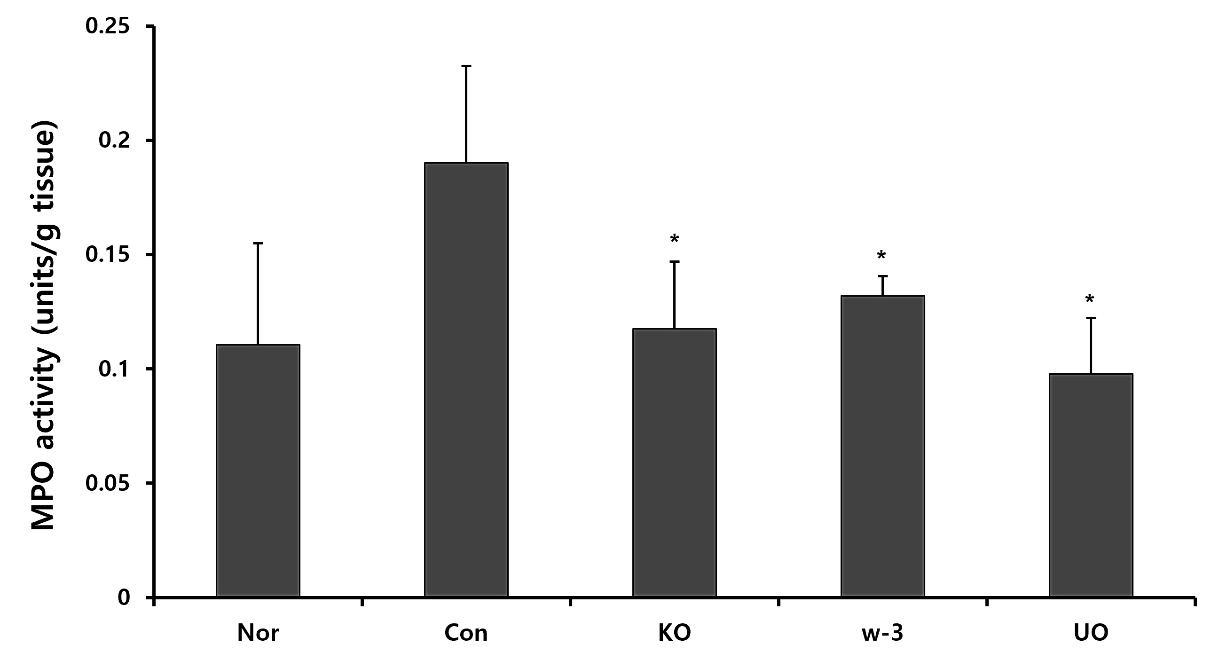


**Fig S4.** MPO activities in colon tissues of each group of mice were determined. Values are presented as the mean ± SE. Means with different letters are significantly different *p<0.05 by Duncan’s multiple range test. Normal group received water without DSS; Control group received 5% DSS in drinking water for 7 days; KO group received 5% DSS and oral administration with krill oil; ω-3 group received 5% DSS and oral administration with omega-3; UO group received 5% DSS and oral administration with *Ulva ohnoi* oil.


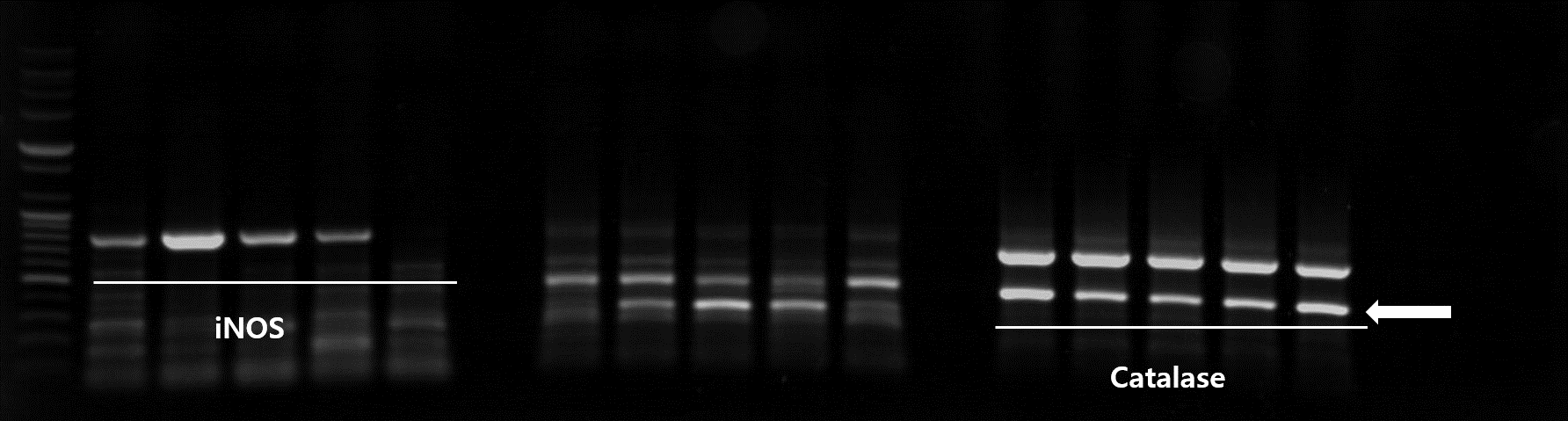


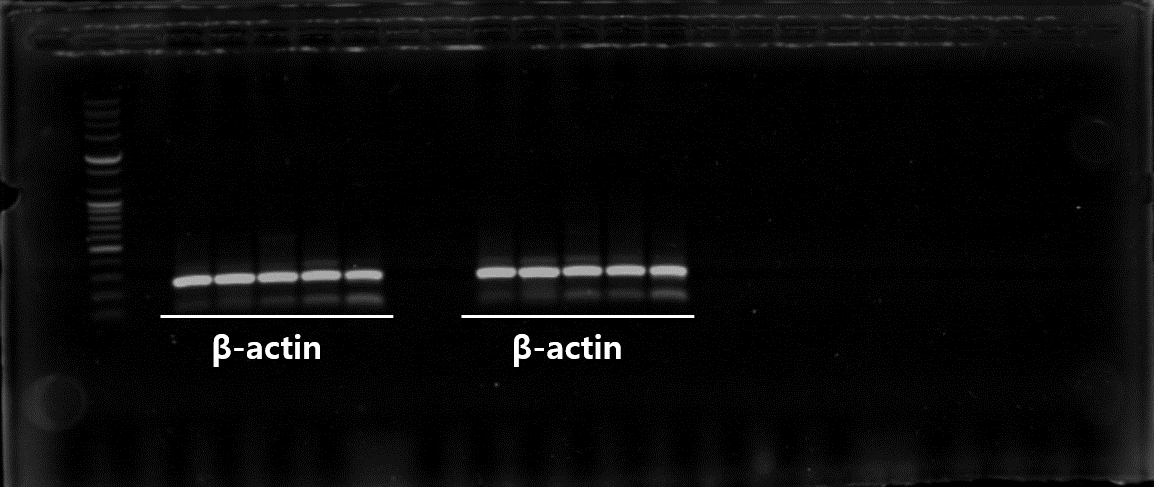


**Fig S5.** Original file for Supplementary Information Fig. S3.
